# Molecular identification of phlebotomine sand flies and the harbored *Leishmania* spp. in Sokoto State, Nigeria

**DOI:** 10.3389/fcimb.2023.1219629

**Published:** 2023-08-31

**Authors:** Mahmud Usman, Audu Joseph Natala, Isa Danladi Jatau, Ndudim Isaac Ogo, Ghulam Jeelani, Yasuyuki Goto, Tomoyoshi Nozaki, James H. McKerrow, Emmanuel Oluwadare Balogun

**Affiliations:** ^1^ Department of Veterinary Parasitology and Entomology, Usmanu Danfodiyo University, Sokoto, Nigeria; ^2^ Department of Veterinary Parasitology and Entomology, Ahmadu Bello University, Zaria, Nigeria; ^3^ Parasitology Division, National Veterinary Research Institute, Vom, Plateau State, Nigeria; ^4^ Department of Biomedical Chemistry, Graduate School of Medicine, The University of Tokyo, Tokyo, Japan; ^5^ Laboratory of Molecular Immunology, Department of Animal Resource Sciences, Graduate School of Agricultural and Life Sciences, Tokyo University, Tokyo, Tokyo, Japan; ^6^ Center for Discovery and Innovation in Parasitic Diseases, Skaggs School of Pharmacy and Pharmaceutical Sciences, University of California San Diego, San Diego, CA, United States; ^7^ Department of Biochemistry, Ahmadu Bello University, Zaria, Nigeria; ^8^ Africa Centre of Excellence for Neglected Tropical Diseases and Forensic Biotechnology, Ahmadu Bello University, Zaria, Nigeria

**Keywords:** Phylogenetics, *Phlebotomus*, *Sergentomyia*, *Leishmania infantum*, Sokoto

## Abstract

**Introduction:**

Female sand flies are hematophagous, feeding on animals and in the process serve as vectors for *Leishmania*, the parasites that cause leishmaniasis in humans. Leishmaniasis are a group of parasitic neglected tropical diseases in 98 countries including Nigeria and kills ~60,000 people/year. In Nigeria, Sokoto State is endemic to leishmaniasis but there is a knowledge gap on the identity of the prevalent sand flies and the *Leishmania* species they transmit. Hence, this cross-sectional study was designed to take inventory of the species of sand flies in Sokoto using genetic methods.

**Methods:**

1,260 (310 females) sand flies were collected from three Local Government Areas (L.G.A) of Sokoto State- Wamakko, Sokoto South and Kware. Genomic DNA was extracted from each fly and DNA amplification by polymerase chain reaction (PCR) was carried out on the DNA samples using primers targeting the arthropods mitochondrial cytochrome oxidase subunit 1 (*mt-coI*) gene, and nested PCR with primers targeting the gene for *Leishmania* internal transcribed spacer-1 (*its-1*) of ribosomal RNA *its-1rRNA*. The PCR products were sequenced.

**Results:**

Gene sequence analysis revealed five species of sand flies belonging to the old-world genera namely *Phlebotomus* and *Sergentomyia*. The identified species were *P. papatasi* (6.45%), *S. adleri* (6.45%), *S. affinis* (9.7%), *S. distincta* (9.7%), *S. schwetzi* (67.7%). Within the sampling period, sand flies were most abundant in the rainy months of August (104/33.5%) and September (116/37.4%) with all the five identified species occurring. Sequence analysis of *its-1* gene identified *Leishmania infantum* in two sand flies (2/310)- *P. papatasi* (from Sokoto South) and *S. affinis* (from Wamakko). BLAST search in NCBI and phylogenetic analysis revealed that the sand fly species are related to the species reported in different parts of Africa, while the *L. infantum* is identical to strain reported in Brazil (KY379083.1).

**Discussion:**

*Phlebotomus papatasi* and four species belonging to the genus *Sergentomyia* are the most prevalent sand flies in Sokoto State, Nigeria and they harbor *L. infantum* solely. The results shed light on why visceral leishmaniasis is the most predominant form of the disease. Therefore, we recommend that adequate care for dogs must be instituted as dogs are the major animal reservoir for *L. infantum*.

## Introduction

Phlebotomine sand flies are the unique hematophagous insects proven to transmit *Leishmania* spp. through the bite of infected female that have previously fed on an infected mammal (Cox, 2002). They are vectors of various pathogenic agents that are responsible for leishmaniasis, bartonellosis and various arboviruses, which are potentially fatal diseases of animals and humans (David et al., 2015). Of these diseases, and from global health viewpoint, leishmaniasis are the most important due to their widespread, morbidities and fatalities in humans (Desjeux, 2004; Nihashi et al., 2016).

Leishmaniasis are caused by protozoan parasites belonging to the genus *Leishmania*, which is further divided into two subgenera- *L.* (*Leishmania)* and L. (*Viannia). Leishmania* protozoa are transmitted through the bite of the female phlebotomine sand fly (Desjeux, 2004). The disease is widely distributed around the world especially in tropical and subtropical areas, affecting at least 12 million people in 98 countries, with an additional 350 million people at risk (Desjeux, 2001). Approximately, twenty (20) *Leishmania* species are known to be pathogenic to humans, and these species are the major determinants of clinical outcome, which are of cutaneous, mucocutaneous, and visceral in nature (Herwaldt, 1999; Choi and Lerner, 2001; Desjeux, 2004).

The spread of leishmaniasis largely depends on the distribution of the vectors, therefore, the identification of circulating sand fly species in endemic and surrounding areas is important for predictions of the risk and expansion of the diseases. Sand flies are generally identified as adults based on morphologic characteristics (Munstermann, 2004). Unfortunately, morphological classification requires considerable skill as well as taxonomic expertise. In addition, the presence of intraspecific variation and cryptic species frequently complicates classifications based on morphological features (Bauzer et al., 2007). Therefore, other characteristics such as molecular markers have been explored for the development of simpler and more accurate identification of sand flies. Several genetic markers have been used to examine the systematics, relationships, and evolution amongst sand fly species and for population analyses within species (Depaquit et al., 2008; Kato et al., 2008; Kuwahara et al., 2009).

The infection of sand flies with *Leishmania* promastigotes has been examined by the dissection of individual sand flies under a microscope. For this purpose, specimens need to be fresh, and the dissection of the sand flies require a highly skilled technique due to their minute size. The procedure takes a relatively long time, and additionally, many specimens must be examined to obtain informative data for each area since the infection rate of sand flies with *Leishmania* is generally very low (0.01–1%) even in endemic areas (Hashiguchi et al., 2003). To improve on conventional methods, several PCR-based techniques which successfully detect the presence of *Leishmania* species within sand flies have been developed (Kato et al., 2005; Kato et al., 2007; Khalid et al., 2010). However, several improvements were desirable for the analysis of many sand flies with less effort and cost. In addition, it is better to analyze sand flies individually because several species co-exist in most endemic areas and the use of pooled samples may compromise important information on vector epidemiology such as the prevalent sand fly species and the relationships between *Leishmania* and vector species (Maroli et al., 2013).

Diagnosis of cutaneous leishmaniasis (CL) in Sokoto state was based on patient clinical presentation and microscopic identification (Jiya et al., 2007; Faleke et al., 2008). Improved identification of the causative *Leishmania* species and their vectors require sophisticated techniques such as molecular biology-based PCR (Schonian et al., 2003; Murray and Cappello, 2008). Molecular-based approaches based on nucleic acids offer greater sensitivity and specificity over the existing diagnostic tests (Cruz et al., 2002; Cruz et al., 2006; Cobo et al., 2007). The techniques permit the detection of infections from very low parasitized samples including those from asymptomatic patients’ samples (Mens et al., 2007). Although DNA-based methods have shown excellent sensitivity and specificity, the introduction of these methods in daily laboratory practice is still uncommon especially in rural endemic regions.

Several molecular markers and polymerase chain reaction (PCR) protocols have been developed for the detection and identification of sand flies and *Leishmania* (Khalid et al., 2010; Falcão de Oliveira et al., 2016). Many of those molecular tools have already been used in different parts of the world to differentiate species (Anderson et al., 2011; Dawit et al., 2013).

## Materials and methods

### Study area

**Figure 1:**
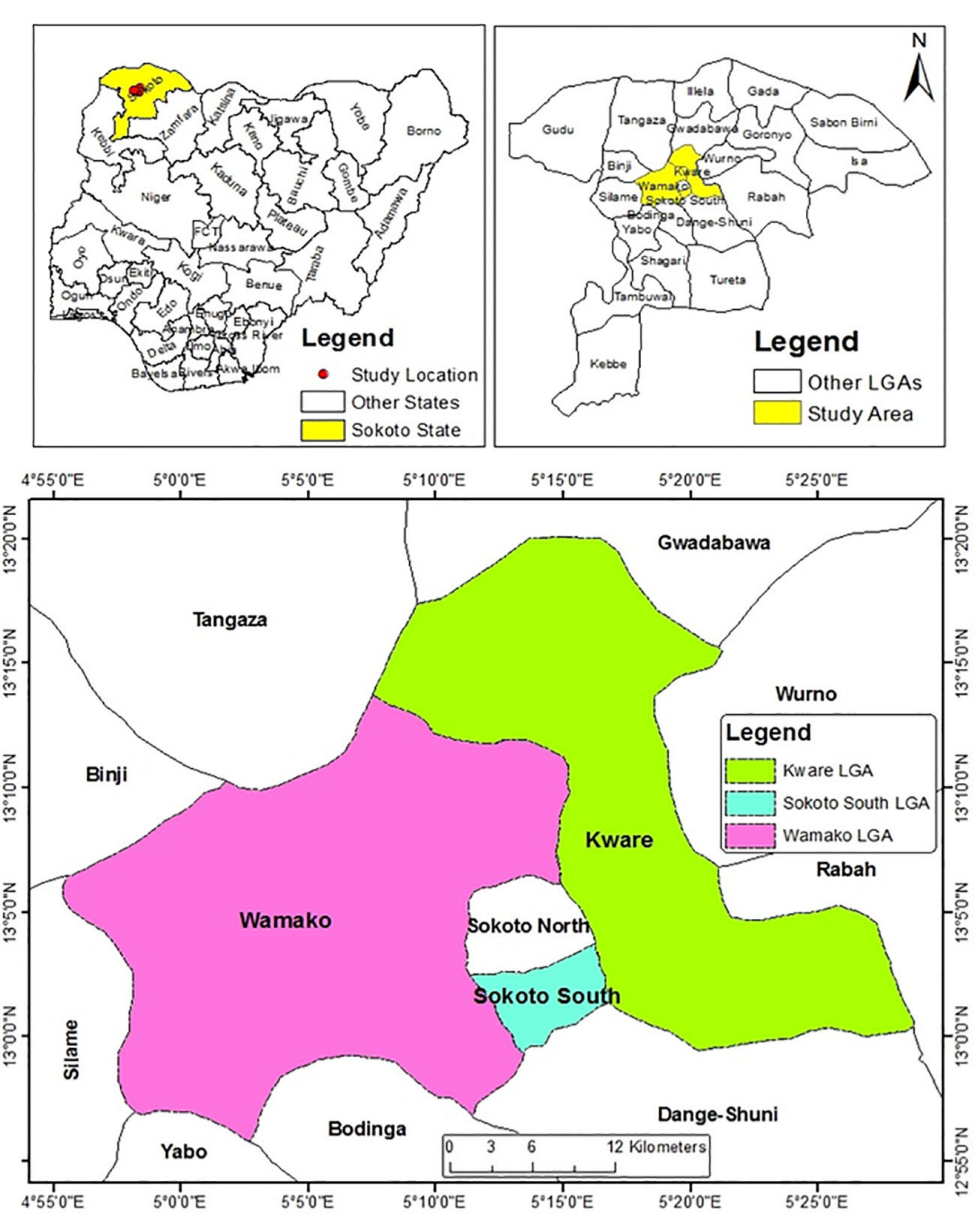
Map of Sokoto State showing the three Local Government Areas Sampled (Source: Department of Geography, A.B.U Zaria).

The study area was Sokoto State, located in the semi-arid region of North-Western Nigeria between longitudes 13° 5' E and 5° 15' E. It shares borders with Niger Republic to the North, Kebbi state to the Southwest and Zamfara State to the Southeast (**Figure 1**). The state has a total land mass of 32,000 km^2^, and is characterized by two distinct seasons, the short rainy season which runs from May or June to September or October and the long dry season that starts from October till May or June (Victor et al., 1997). The minimum relative humidity is less than 20% for most part of the year and ambient temperature ranges from 22 °C to 43 °C.

### Study design

This cross-sectional study covered three Local Government Areas of Sokoto state.

Sand fly habitats were identified based on reported cases of human cutaneous leishmaniasis within the study area.

### Collection of sand flies and morphological identification

Survey was carried out between May and November 2016 (Usman et al., 2020a). Sand flies were collected using improvised sticky traps made of plywood (25 × 40 cm) coated on both sides with engine oil and kept around sand fly breeding sites onto which randomly impinging sand flies adhered. Traps were set thrice weekly. During each trapping session, ten (10) traps were allocated to each site (Refuse dumps and Sewage tanks). Traps were set between 17:30 and 18:30 hr and collected between 06:00 and 07:00 hr of the following day. In the laboratory, sand flies were removed from the sticky traps using dissecting needle, washed in kerosene, soapy water, then clean water (to reduce viscosity), and observed under the microscope for sex identification (Usman et al., 2020a). The female flies were transferred into tubes containing 70% ethanol and stored at 4 °C.

Morphological identification of sand fly specimens was carried out using Kyowa Stereo microscope HWFX (Kyowa, Tokyo, Japan) under the 10× eye pieces and using the established standard keys of Abonnenc (1972) and Lewis (1982). Sand flies were morphologically identified using body size and shape of wings. The presence or absence of external genitalia was used to identify categorize as male or female sand fly (Young and Duncan, 1994).

### DNA extraction

Genomic DNA was extracted from each of 400 morphologically identified female sand flies using Speedtools Tissue DNA extraction Kit (Biotools B&M Labs, Madrid, Spain). Briefly, each fly was washed with 200 µL distilled water to remove residual ethanol and thoroughly homogenized with a pestle in a laminar air flow. Exactly 130 µL of buffer ATL was added to each sample tube followed by 20 µL of proteinase K and mixed thoroughly by vortexing before incubation at 56 °C overnight in a heating block. The subsequent steps were carried out exactly according to the manufacturer’s guide. The eluted DNA was transferred into 1.5 mL tube with screw cap. Samples were incubated for 15 minutes at 100 °C to inactivate residual proteinase K before storage at -20 °C until required for PCR. The extracted DNA is expected to contain either the gDNA of sand flies alone or a mixture of the gDNA of sand flies and harbored pathogens such as *Leishmania* spp.

### Polymerase chain reaction for amplification of diagnostic genes of sand flies

A summary of the PCR conditions and primers used for amplification of the indicated target genes for the Leishmania, and sand flies is provided in “**Supplementary Table 1**”. Owing to the genus and species specificity of the arthropod mitochondrial cytochrome C oxidase sub-unit 1 gene (*mtcoI*), it is generally targeted for amplification and sequencing, for the purpose of molecular identification and characterization. Therefore, *mtcoI* was amplified in a reaction containing 100 ng of the template DNA, 2.5 µM of the forward (5'-GGTCAACAAATCATAAAGATATTGG-3') and reverse (5'-TAAACTTCAGGGTGACCAAAAAATCA-3') primers, 1× of PCR reaction buffer containing 2 mM MgCl_2,_ 250 µM of each dNTPs, and 1.0 U of PfuUltra II Fusion HS DNA Polymerase (Agilent technologies, Santa Clara, CA, USA) all in a total reaction volume of 50 µL. The negative controls were devoid only of DNA samples. Tubes were placed in a thermocycler and the program was run according to protocol by Hebert et al. (2003). The PCR cycling conditions were initial denaturation at 94 °C for 5 mins, 35 cycles of denaturation at 94 °C for 30 seconds, annealing at 55 °C for 1 min, and extension at 72 °C for 1 min, followed by final extension at 72 °C for 10 mins. The PCR products were stored at −20 °C until required.

For diagnosis and species identification, two *Leishmania* genes- small sub-unit ribosomal RNA *(ssu rRNA)* and ribosomal internal transcribed spacer-1 *(its-1)* were targeted for amplification of the species-specific portions by nested PCR (nPCR). The first PCR for amplification of the full length *ssurRNA* and *its-1* were done using the primer pairs (forward: 5'-GGTTCCTTTCCTGATTTACG-3' and reverse: 5'-GGCCGGTAAAGGCCGAATAG-3') and (forward: 5'-CTGGATCATTTTCCGATG-3' and reverse: 5'-TGATACCACTTATCGCACTT-3'), respectively. Twenty five microliter reaction mixtures were prepared containing 50 ng of DNA, 0.5 U of PfuUltra II Fusion HS DNA Polymerase (Agilent technologies, Santa Clara, CA, USA), 1× of reaction buffer containing 2 mM MgCl_2,_ 250 µM of each dNTPs, and 2.5 µM of the respective primers.

The PCR cycling protocol involved initial denaturation at 94 °C for 5 mins, 30 cycles of denaturation at 94 °C for 30 secs, annealing at 60 °C for 30 secs, and extension at 72 °C for 30secs. Final extension was performed at 72 °C for 5 mins. The PCR products were stored at −20 °C until required for the second PCR.

The second PCR of the nested technique to amplify the internal portions both *ssu rRNA* and *its-1* was performed in using the same reaction constituents as the first step except that the DNA template was 5 µL product of the first PCR, and the primers (forward: 5'-TCCCATCGCAACCTCGGTT-3' and reverse: 5'-AAAGCGGGCGCGGTGCTG-3') and (forward: 5'-CATTTTCCGATGATTACACC-3' and reverse: 5'-CGTTCTTCAACGAAATAGG-3'), respectively, were used. The PCR cycling conditions were initial denaturation at 94 °C for 5 mins, 30 cycles of denaturation at 94 °C for 30 secs, annealing at 65 °C for 30 secs, and extension at 72 °C for 10 secs. Final extension was performed at 72 °C for 1min. The PCR products were stored at -20 °C until required for electrophoresis and sequencing.

### Agarose gel electrophoresis and gel purification of PCR products

Agarose (1.5%) was melted in 1× TAE buffer containing SYBR Safe and polymerized in gel tray. Samples were prepared by addition of 10× loading dye, loaded into agarose gel wells alongside 100 bp DNA ladder, and electrophoresed at 80 V for 30 minutes.. The Gels were visualized under UV light and the images were documented. Each DNA band containing the expected specific amplicon was carefully excised using a scalpel blade and purified using QIAquick Gel Extraction Kit (Qiagen, Hilden, Germany) according to manufacturer’s protocol.

### Sequencing and phylogenetic analysis

Sequencing primers were designed based on *mtcoI* sequences (for sand flies) and *its-1* sequences (for *Leishmania*) that were obtained from the GenBank. The gel-purified PCR products were sequenced using the BigDye Terminator v3.1 cycle sequencing kit (Applied Biosystems, Carlsbad, CA). Data were analyzed with ABI 3130 genetic analyzer software.

The sequence chromatograms were viewed and edited using ApE genetic analyzer. Each sequence was imported into the NCBI Database and nucleotide Basic Local Alignment Search Tool (BLAST) was used to search for similarity with other sequences in the GenBank using the NCBI database search (https://www.ncbi.nlm.nih.gov/BLAST).

The evolutionary relationship of sand flies and *Leishmania* specie isolates were determined by the construction of a phylogenetic tree using the Molecular Evolutionary Genetic Analysis (MEGA 7.0) software (Kumar et al., 2016). All species were separated with each one having its own branch. Sequences for all newly collected isolates clustered together with those already published for the respective species of the same or different locality forming groups of the same sub genus. The phylogenetic groupings provided by the tree, coupled with the aforementioned sequencing queries against GenBank, confirmed the molecular and morphological identification of the sampled sand flies and *Leishmania* species.

### Data analysis

Data collected was presented as tables, figures and plates (SPSS Version 20). Values of P < 0.05 were considered significant. Bioclimatic data, including relative humidity, rainfall and average temperature, were obtained from the Nigerian Meteorological Agency as reported by https://www.timeanddate.com/weather/nigeria/sokoto for each month of the study period.

## Results

### Identification of sand flies and *Leishmania*

**Figure 2:**
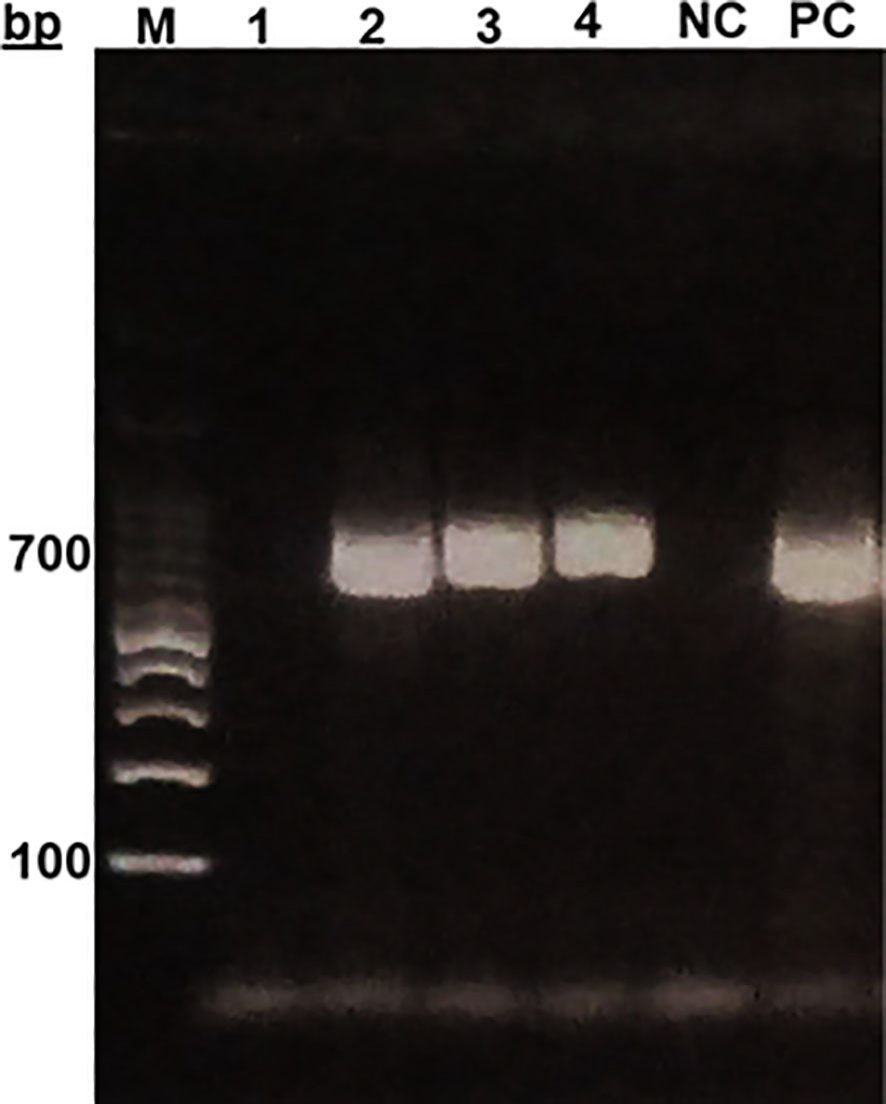
Gel electrophoresis of *mt-co1* of sand flies from Sokoto. MM- Molecular marker (100bp DNA ladder) PC (positive control), NC (Negative control).

**Figure 3:**
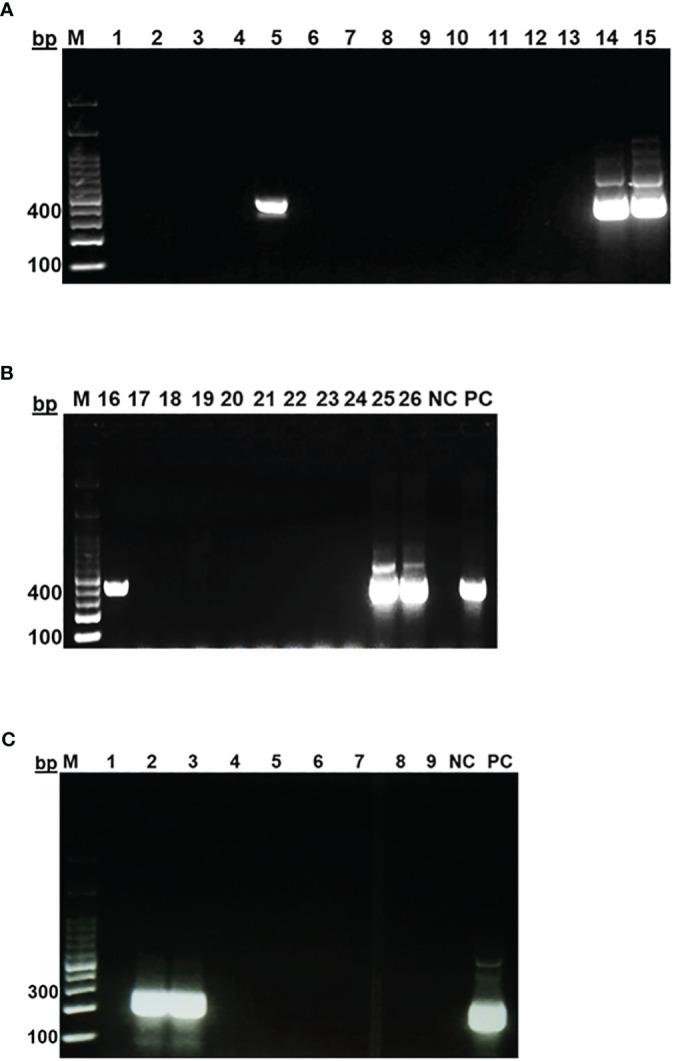
Identification of *Leishmania* spp. in female sand flies in Sokoto. Gel electrophoresis of PCR products targeting *ssu rRNA* gene **(A, B)** and *its-1* gene **(C)**. MM- Molecular marker (100bp DNA ladder), NC (Negative control), PC (Positive control).

In general, sand flies are small with relatively long legs and erect wings as seen. While for *Phlebotomus* species, wings are asymmetrical and erect tapering towards the end and close to the body as observed while, *Sergentomyia* species the wings are symmetrical. While identification of the sand flies and *Leishmania* genera are possible by microscopic examinations, accurate identification and categorization into their species can only be confirmed using molecular biology tools involving PCR and DNA sequencing. Trap-captured female sand flies were identified microscopically and counted, and then DNA were prepared from the whole individual fly. The extracted DNA, which may also contain DNA of *Leishmania*, were subjected to two different nPCR for molecular identification of the sand flies and confirmation of the presence or absence of *Leishmania* parasites. The nPCR assays amplified the 700 bp segment of fly’s *mtcoI* (**Figure 2**), and the targeted 400 bp and 280 bp segments of *ssurRNA* and *its-1*, respectively, in DNA sample of a *Leishmania* positive sand fly (**Figures 3A–C**).

### Genotypes of sand flies and *Leishmania*

**Figure 4:**
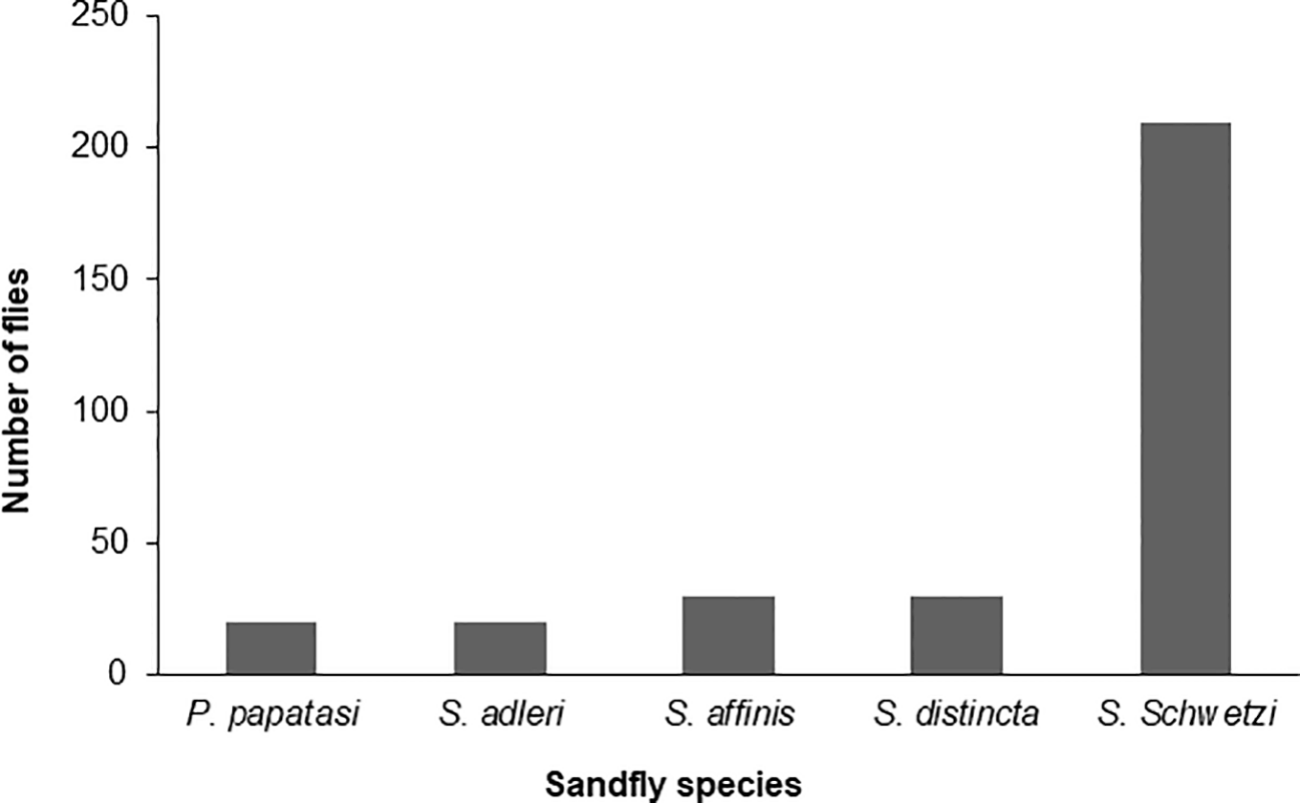
Species of sand fly in different parts of Sokoto State.

**Figure 5:**
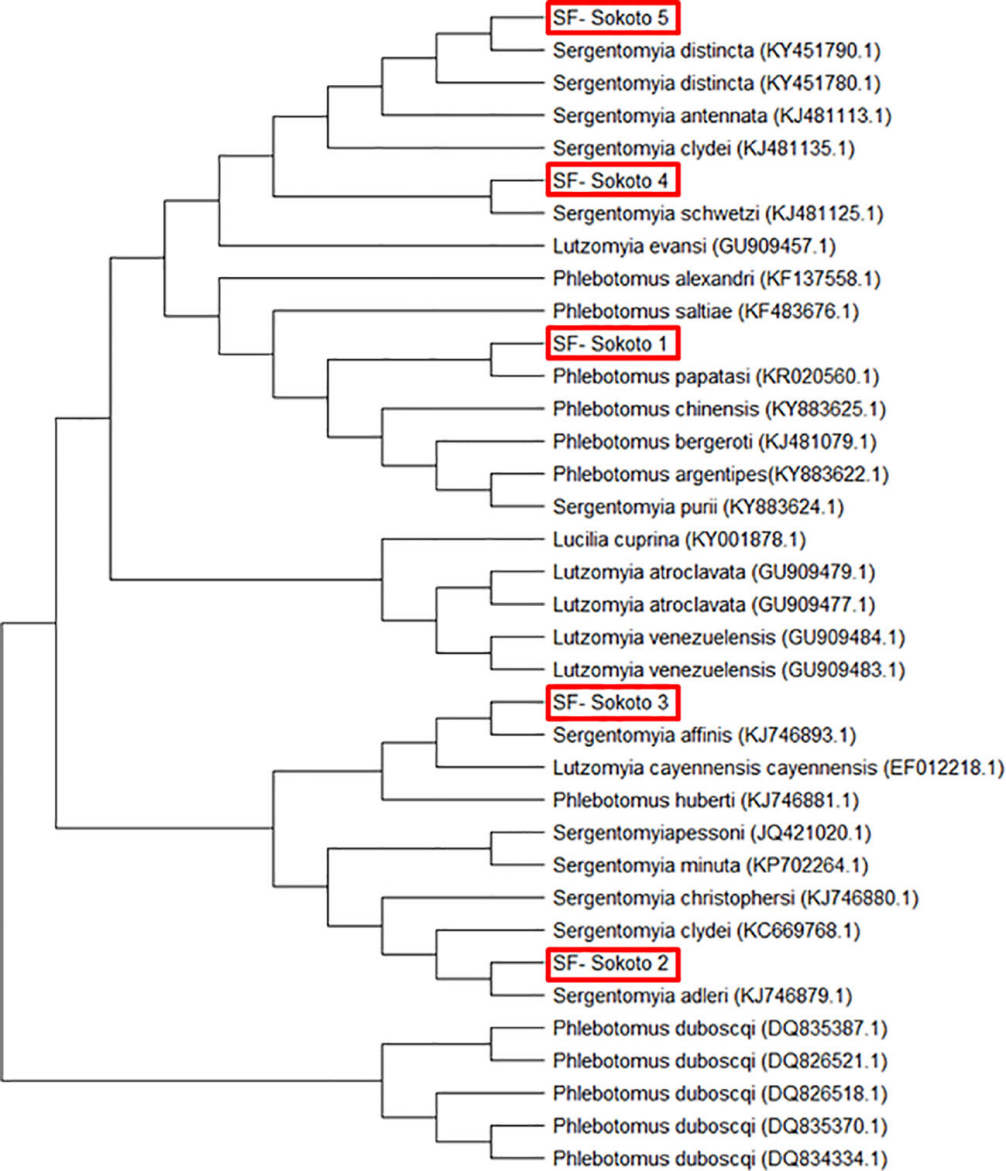
Phylogenetic tree of sand fly species from Sokoto State with other sand fly species sequences obtained from GenBank using MEGA 7.0.

*Sand flies*: Amplification of the *mtcoI* gene was carried out by nPCR on DNA samples prepared from 400 female sand flies. Sequence analysis of the 400 PCR products revealed that they clustered into five different consensus nucleotide sequences- SF-Sokoto 1, SF-Sokoto 2, SF-Sokoto 3, SF-Sokoto 4, and SF-Sokoto 5). BLAST search with each consensus sequence revealed that SF-Sokoto_1 was 99% identical to *Phlebotomus papatasi* (KR 020560.1), SF-Sokoto 2 was 98% identical to *Sergentomyia adleri* (KJ746879.1), SF-Sokoto 3 was 99% identical to *S. affinis* (KJ746893.1), SF-Sokoto 4 was 100% identical to *S. schwetzi* (KJ481125.1), while SF-Sokoto 5 was 100% identical to *S. distincta* (KY451790.1). Our results imply that the sand fly population of Sokoto, Nigeria are comprised mainly of *P. papatasi*, *S. adleri, S. affinis, S. distincta, and S. schwetzi*. These species of sand flies constitute (20%, 6.5%, 10%, 10%, 67%) of the sampled flies in Sokoto state respectively (**Figures 4**, **5**).

**Figure 6:**
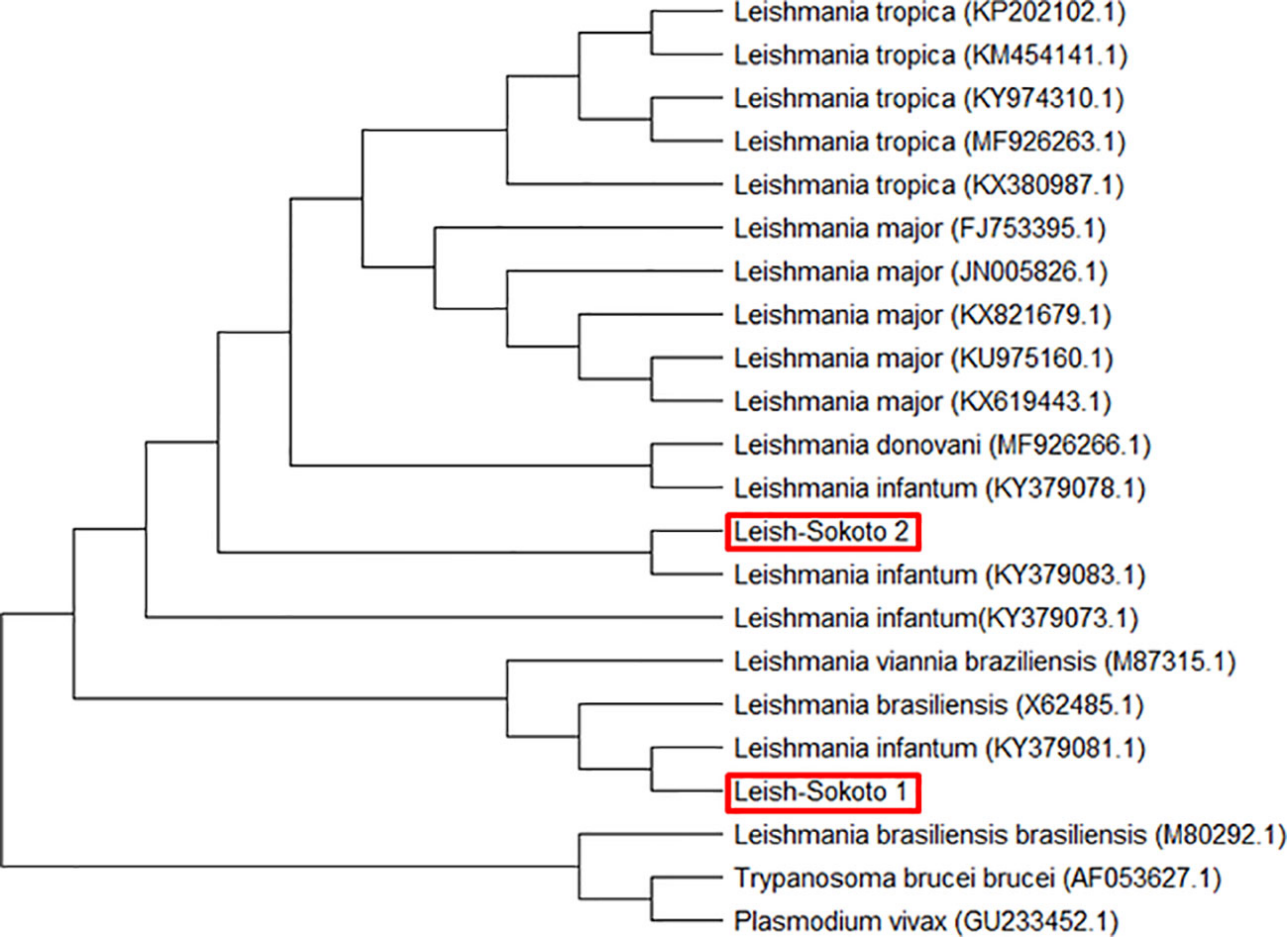
Evolutionary relationship of *Leishmania infantum* from Sokoto State with other similar isolates from different localities based on ITS-1 gene constructed using MEGA 7.0.

*Leishmania species*: Sequence analyses of the targeted inner segment of Leishmania its-1 for the 400 DNA samples that were prepared from female sand flies revealed 2 different but related consensus nucleotide sequences– Leish-Sokoto 1 and Leish-Sokoto 2. The sequences have been deposited to the GenBank, with accession codes MN243118.1 and MN243117.1, respectively. These sequences were used to search the GenBank (blastn; http://blast.ncbi.nlm.nih.gov). Both Leish-Sokoto 1 and 2 were identified as *Leishmania infantum*, with 98% and 100% identities with *L infantum* isolate from Brazil (KY379083.1) and Greece (KY379081.1), respectively (**Figure 6**).

### Occurrence of sand fly species in the 3 locations of Sokoto State

While all the reported 5 sand fly species were found in Wamakko L.G.A., (occurrence rates of 5.3%, *P. papatasi*; 5.3%, *S. adleri*; 15.7%, *S. affinis*; 10.5%, *S. distincta*; *63.2%*, *S. schwetzi*), only 4 species were captured in Sokoto-South L.G.A., (occurrence rates: 8.3%, *P. papatasi*; 8.3%, *S. adleri*; 8.3%, *S. distincta*; 75.0%, *S. schwetzi*). *S. affinis* was absent in Sokoto-South, and no sand fly was captured in Kware L.G.A (**Table 1**).

**Table 1:** Occurrence of female sand fly species from 3 Locations in Sokoto State.

Location	*P. papatasi* *n (%)*	*S. adleri* *n (%)*	*S. affinis* *n (%)*	*S. distincta* *n (%)*	*S.schwetzi* *n (%)*	*Total* *n(%)*
Wamakko	10 (5.3)	10 (5.3)	30 (15.7)	20 (10.5)	120 (63.2)	190 (100.0)
Sokoto South	10 (8.3)	10 (8.3)	0 (0.0)	10 (8.3)	90 (75.0)	120 (100.0)
Kware	0 (0.0)	0 (0.0)	0 (0.0)	0 (0.0)	0 (0.0)	0 (0.0)

### *Leishmania*-habouring sand fly species in Sokoto State 

The nPCR detected *Leishmania* DNA in samples prepared from *P. papatasi* from Sokoto South and *S. affinis* from Wamakko. DNA sequencing identified the *Leishmania* as *L. infantum* (**Table 2**). Out of the twenty *P. papatasi* species identified, 5% were positive for *L. infantum* DNA, and one out of 30 *S. affinis* species was positive for *L. infantum* (3%). This is the first time we are recording the presence of *Leishmania* DNA in *Sergentomyia* species in Nigeria. Other species (*S. distincta, S. adleri and S. schwetzi)* were negative for *Leishmania* DNA. (**Table 2**). Only 310 out of the 400 PCR products from female sand flies produced readable sequences after direct sequencing, although DNA quantity was not a limiting factor. Total infection rate of *Leishmania* in sand flies, in both Sokoto South and Wamakko L.G.A of Sokoto State was 0.6% (2/310) and this is epidemiologically significant.

**Table 2:** Identified *Leishmania Species* in Sand flies by Sequence Analysis of ITS-1 gene.

Sand fly Species	No of samples	*Leishmania* +ve n (%)	*Leishmania* species	Location
*P. papatasi*	20	1 (5.0)	*L. infantum*	Sokoto South
*S. affinis*	30	1 (3.0)	*L. infantum*	Wamakko
*S. adleri*	20	0 (0.0)	_	_
*S. distinct*	30	0 (0.0)	_	_
*S. schwetzi*	210	0 (0.0)	_	_
Total	310	2 (0.6)	_	_

### Monthly occurrence of sand fly species

Of the five (5) species of sand flies identified, three (3) were reported in the dry months of May; *S. affinis* (33.3%), *S. distincta* (16.7%) and *S. schwetzi* (50%) and in November only *S. schwetzi* (100%) was found. All the 5 species were reported in the rainy months of August; *P. papatasi* (3.8%), *S. adleri* (1.0%), *S. affinis* (19.2%), *S. distincta* (16.4%), *S. schwetzi* (59.6%) and in September; *P. papatasi* (9.5%), *S. adleri (16.4%), S. affinis* (1.7%), *S. distincta* (6.0%), *S. schwetzi* (66.4%) (**Table 3**). These results imply that irrespective of the season, *S. schwetzi* is the dominant sand fly species, and *P. papatasi* is the least prevalent and found only during the rainy season. Overall, the sand flies of the *Sergentomyia* genus account for between 80-100% of the sand fly population in Sokoto State.

**Table 3:** Monthly occurrence of different sand fly species in Sokoto State.

Months	*P. papatasi*	*S. adleri*	*S. affinis*	*S. distincta*	*S. schwetzi*	Total
May	0 (0.0)	0 (0.0)	4 (33.3)	2 (16.7)	6 (50.0)	12 (100.0)
June	0 (0.0)	0 (0.0)	0 (0.0)	0 (0.0)	10 (100.0)	10 (100.0)
July	0 (0.0)	0 (0.0)	4 (11.8)	4 (11.8)	26 (76.4)	34 (100.0)
August	4 (3.8)	1 (1.0)	20 (19.2)	17 (16.4)	62 (59.6)	104 (100.0)
September	11 (9.5)	19 (16.4)	2 (1.7)	7 (6.0)	77 (66.4)	116 (100.0)
October	5 (16.7)	0 (0.0)	0 (0.0)	0 (0.0)	25 (83.3)	30 (100.0)
November	0 (0.0)	0 (0.0)	0 (0.0)	0 (0.0)	4 (100.0)	4 (100.0)

### Phylogenetic analysis for sand flies

Based on the estimated molecular size of the PCR products, five species of sand flies were identified at the study location. The five sand fly (SF) samples were code-named SF-Sokoto 1, SF-Sokoto 2, SF-Sokoto 3, SF-Sokoto 4, and SF-Sokoto 5. The evolutionary relationship of the sand fly species is illustrated as a phylogenetic tree (**Figure 5**). Phylogenetic analysis revealed that all the five representative flies are of 2 different genera and belong to five different species namely, *Phlebotomus papatasi*, *Sergentomyia adheleri*, *S. affinis*, *S. schwetzi*, and *S, distincta*, respectively (**Figure 5**). Sequences for all newly collected isolates formed five different clusters with those already published for the respective species from Africa. The phylogenetic groupings provided by the tree, coupled with the aforementioned sequencing queries against GenBank confirmed the molecular and morphological identification of the sampled sand flies.

### Phylogenetic analysis for *Leishmania* species

Phylogenetic analysis using the *ssu rRNA* nucleotide sequences showed two categories of sequences that are pure clusters with each other and with those of *L. infantum* (KY379073.1, KY379078.1, KY379081.1, KY379083.1) isolates from Europe (**Figure 6**). The sequences from this study were those from the parasite isolates from Sokoto, code-named Leish-Sokoto 1 and Leish-Sokoto 2 (**Figure 6**). The evolutionary groupings provided by the tree, coupled with the sequencing queries against GenBank, confirmed the molecular identification of the *Leishmania* species present in the sampled sand flies as *L. infantum*.

## Discussion

Leishmaniasis is an important emerging parasitic disease found in 98 countries around the world (World Health Organization, 2010), and domestic dogs are the principal reservoir hosts and while wild canids constitute major sylvatic reservoirs (Ashford, 2000). Intense transmission of leishmaniasis by infected sand flies, from dog to dog or from dog to human, occurs in places where the *Leishmania* infection rate is very high in dogs (Vercammen et al., 1997). Sokoto State is an endemic focus for leishmaniasis in Nigeria. A 3.5% seroprevalence of canine leishmaniasis was reported in Sokoto State (Usman et al., 2020b). Jiya et al. (2007) reported a 10% prevalence of cutaneous leishmaniasis in school children in the study area, while Faleke et al. (2008) published a case report of cutaneous leishmaniasis in an undergraduate university student in Sokoto State. However, none of the reported cases adopted molecular techniques in the diagnosis of the disease. Visceral leishmaniasis is not routinely diagnosed in west Africa (Coulibaly et al., 2016). This is the first molecular studies on phlebotomine sand flies *Leishmania* species in the study area. We previously established by morphological observation that male sand fly population in Sokoto is twice the female population (Usman et al., 2020a) but there is no information on the prevalent parasite species as well as the vector species. In this study, we identified five species of phlebotomine sand flies as the potential vectors for leishmaniasis in Sakoto State, Nigeria. Of which sand flies belonging to the old-world genera namely *Phlebotomus* (Rondani, 1840) and *Sergentomyia* (França and Parrot, 1920) were identified: One (1) *Phlebotomus* spp., (*P. papatasi*) and four *Sergentomyia* spp. (*S. adleri, S. affinis, S. distincta and S. schwetzi*). The preponderance of *Sergentomyia* spp. over *Phlebotomus* spp. is similar to earlier findings in Cameroon (Rageau, 1951; Rageau and Adam, 1953), some parts of Nigeria (Agwale et al., 1995; Adamu et al., 2012) and in The Gambia (Desjeux et al., 1983).

*Sergentomyia schwetzi* was the most abundant species and it has been found in all the study areas. This is similar to the findings of Sangare et al. (2009) in Burkina Faso, Dondji et al. (2000) in Cameroon where *S. schwetzi* was found to be the most abundant species. The other dominant sand flies of the genus *Sergentomyia* were *S. distincta* and *S. affinis*. *P. papatasi and S. adleri* are the third most abundant species, both were recorded in Sokoto South and Wamakko. In this study, only the areas of suspected transmission were investigated. Moreover, *P. papatasi* was the only species identified from the genus *Phlebotomus*. For this reason, this species could be considered as the probable main vector of *Leishmania* spp. parasites. Until now, *P. duboscqi* was reported to be the principal vector of *Leishmania* in Nigeria (Killick-Kendrick, 1990). Our present findings on the skewed occurrence of *P. papatasi* in Sokoto State, which also coincided with prevalence of *Leishmania* infections in animals in the same location suggests that *P. papatasi* is also a major player in the transmission of leishmaniasis in Nigeria. Furthermore, although *P. papatasi* is the recognized vector of *L. major* in the old world (Anis et al., 2001), the results herein also implicate *P. papatasi* may be involved in the transmission of *L. infantum* corroborating that the multiple sand fly species may be serving as the vectors for *L. infantum*. We recommend that the vectoral capacity of *P. papatasi* for transmission of *L. infantum* should be investigated.

The natural occurrence of *Leishmania* parasites in sand flies is an important determinant of active transmission in a particular locality (Ready, 2013). Therefore, it is important to conduct routine surveillance of parasites in the midgut of sand flies. Dissection of sand fly gut is the gold-standard method used to study the rate of natural infection in endemic areas. This method is laborious, time consuming and requires a lot of skills and expertise. It also requires a large number of specimens to achieve reasonable epidemiological data (Aransay et al., 2000). Alternatively, molecular techniques allow for DNA detection of a single *Leishmania* parasite (Pita-Pereira et al., 2005) and probably represent a more sensitive tool than manual dissection and microscopic examination (Nascimento et al., 2007), which may underestimate natural sand fly infection rates in cases of low parasitemia. The Polymerase chain reaction (PCR) is a suitable technique for the detection of *Leishmania* DNA in sand flies and for identification of *Leishmania* vectors in different geographical areas (Aransay et al., 2000). Molecular methods are more sensitive and specific, regardless of the number, stage, and location of the parasite in the insect midgut (Perez et al., 1994). The PCR technique was therefore used in this study.

From literature, the role of *Sergentomyia* spp. in the circulation of *Leishmania* spp. is becoming more apparent as *Leishmania* DNA has been identified in several species of the genus. These include the molecular detection of *L. major* in *S. sintoni* in Iran (Parvizi and Amirkhani, 2008), *S. garnhami* in Kenya (Mutinga et al., 1994), *S. darlingi* in Mali (Berdjane-Brouk et al., 2012), and *S. minuta* in Portugal (Campino et al., 2013). Furthermore *L. donovani* has been detected in *S. babu* in India (Mukherjee et al., 1997), *L. infantum* in *S. dubia*, *S. magna* and *S. schewtzi* in Senegal (Senghor et al., 2011) and more recently, *L. tropica* has been found in *S. ingrami* and *S. hamoni* in Ghana (Nzelu et al., 2014). Although *L. infantum* had been detected in *S. schwetzi* from Senegal (Senghor et al., 2011), the refractoriness of this African species to some *Leishmania* species infecting humans (including *L. donovani, L. infantum* and *L. major*) has also been recently demonstrated (Sadlova et al., 2013). This study is the first to detect the presence of *Leishmania* sp. (*L. infantum*) in *Sergentomyia* species (*S. affinis*) in Nigeria, which is similar to findings from other parts of the world as previously discussed. This further confirms the possibility of *Sergentomyia* species becoming a vector of *Leishmania* parasites, hence should be of great relevance in the epidemiology of leishmaniasis in Nigeria and other parts of Africa. Worthy of mention, according to literature the occurrence of sand flies in the rainy season is uncommon. However, the rainy season in Sokoto State is very short (about 47.17 rainy days in a year) and precipitation is as low as 34.38 mm. In addition, the average daily mean temperature can be as high as 39 °C. These climatic conditions could be the reasons for the presence of sand flies even in the rainy season in Sokoto state.

*Leishmania infantum* is the causative agent of infantile visceral leishmaniasis in the Mediterranean region of the Old World and in Latin America, where it has been named *Leishmania chagasi*. (Maurício et al., 2000). It is also an unusual cause of cutaneous leishmaniasis (BenSaid et al., 2006). *Leishmania infantum* is closely related to *L. donovani*, and some authors believe that these two species are so close as to be subspecies of each other; (Le Blancq and Peters, 1986) however, phylogenetic analyses can easily distinguish between the two groups, although analysis has shown that some isolates of *L. donovani* have been classified as *L. infantum* and that the former includes a number of different genetic groups (Kuhls et al., 2005). *P. papatasi* supports the development of only *L. major* (Killick-Kendrick et al., 1994). However, other sand fly species support the development of wider range of *Leishmania* spp. The detection of phylogenetically and epidemiologically distant species of *L. infantum* could be due to natural genetic hybridization between *L. major* and *L. infantum* as reported by Ravel et al. (2006) and Volf et al. (2007). The reports raise questions about the frequency of such cross species genetic exchanges in nature, modalities of hybrid transmission, and their long-term maintenance as well as consequences of the genetic hybrids. Human infection with *L. infantum* is zoonotic with dogs serving as the reservoir hosts. Interestingly, Usman et al. (2020b) reported a 3.5% seroprevalence of canine leishmaniasis in the study area. This report may explain the possible existence of *L. infantum* in Sokoto State, Nigeria.

Visceral leishmaniasis is not routinely diagnosed in West Africa (Coulibaly et al., 2016). For the first time, the occurrence of *L. infantum* in Sokoto State Nigeria was observed in two species of sand flies (*P. papatasi and S. affinis*). It is possible that clinicians are misdiagnosing cases of this disease and confusing symptoms with malaria, toxoplasmosis, or another infectious fever. Therefore, the detection of *L. infantum* in the study area is of great public health significance.

## Conclusion

Five species of phlebotomine sand flies belonging to the Old-World genera namely *Phlebotomus* (Rodani and Berte, in Rodani 1840) and *Sergentomyia* (França and Parrot, 1920) were identified: One *Phlebotomus* sp., (*P. papatasi*) and four *Sergentomyia* spp. (*S. adleri, S. affinis, S. distincta and S. schwetzi*) were detected. The LnPCR detected *Leishmania* DNA in two (2) sand fly species (0.6%) belonging to *P. papatasi* from Sokoto South and *S. affinis* from Wamakko, out of the 310 female sand flies analyzed. Bearing in mind the contribution of climate change to thriving of disease vectors (Balogun et al., 2016; Adepoju et al., 2023), it is important to increase surveillance efforts for vectors of parasitic infections. The outcomes may further stimulate efforts towards the discovery of new drugs (Ungogo et al., 2020).

## Data availability statement

The data presented in the study are deposited in the https://www.ncbi.nlm.nih.gov/nuccore repository, with accession numbers MN243117.1 and MN243118.1.

## Author contributions

MU, AN and EB conceptualized the project. AN, IJ, and EB supervised the work. MU, NO, GJ, YG, and EB contributed to the development and writing of the manuscript. YG, TN, JM and EB contributed to validating and reviewing the project. All authors contributed to the article and approved the submitted version.
