# Supplementary material for: Molecular identification of phlebotomine sand flies and the harbored Leishmania spp. in Sokoto State, Nigeria
Source: Front Cell Infect Microbiol. 2023 Aug 31;13:1219629. doi: 10.3389/fcimb.2023.1219629 (PMC10500309; doi:10.3389/fcimb.2023.1219629)
Supplement: Supplementary file 1 [file Table_1.docx]

| Target gene | Primer sequence (5' → 3') | PCR condition |
| --- | --- | --- |
| **Sand fly** |  |  |
| *mtcoI* | GGTCAACAAATCATAAAGATATTGG | 94 °C (5 min) |
|  | TAAACTTCAGGGTGACCAAAAAATCA | **35 cycles** |
|  |  | 94 °C (30 s) |
|  |  | 55 °C (1 min) |
|  |  | 72 °C (1 min) |
|  |  | 72 °C (10 min) |
|  |  | 4 °C (∞) |
| **Leishmania** |  |  |
| *ssurRNA* | GGTTCCTTTCCTGATTTACG | 94 °C (5 min) |
| first nested | GGCCGGTAAAGGCCGAATAG | **30 cycles** |
|  |  | 94 °C (30 s) |
|  |  | 60 °C (30 s) |
|  |  | 72 °C (30 s) |
|  |  | 72 °C (5 min) |
|  |  | 4 °C (∞) |
| *its-1* | CTGGATCATTTTCCGATG | 94 °C (5 min) |
| first nested | TGATACCACTTATCGCACTT | **30 cycles** |
|  |  | 94 °C (30 s) |
|  |  | 60 °C (30 s) |
|  |  | 72 °C (30 s) |
|  |  | 72 °C (5 min) |
|  |  | 4 °C (∞) |
| *ssurRNA* | TCCCATCGCAACCTCGGTT | 94 °C (5 min) |
| second nested | AAAGCGGGCGCGGTGCTG | **30 cycles** |
|  |  | 94 °C (30 s) |
|  |  | 65 °C (30 s) |
|  |  | 72 °C (10 s) |
|  |  | 72 °C (1 min) |
|  |  | 4 °C (∞) |
| *its-1* | CATTTTCCGATGATTACACC | 94 °C (5 min) |
| second nested | CGTTCTTCAACGAAATAGG | **30 cycles** |
|  |  | 94 °C (30 s) |
|  |  | 60 °C (30 s) |
|  |  | 72 °C (30 s) |
|  |  | 72 °C (5 min)  4 °C (∞) |

**Supplementary Table 1:** PCR conditions for identification of sand flies and *Leishmania* spp.
